# Spousal Presence as a Nonpharmacological Pain Management during Childbirth: A Pilot Study

**DOI:** 10.1155/2015/932763

**Published:** 2015-11-22

**Authors:** Abigail U. Emelonye, Taina Pitkäaho, Katri Vehviläinen-Julkunen

**Affiliations:** ^1^Department of Nursing Science, Faculty of Health Sciences, University of Eastern Finland, Yliopistonranta 1C, P.O. Box 1627, 70211 Kuopio, Finland; ^2^Kuopio University Hospital (KUH), P.O. Box 100, 70029 Kuopio, Finland

## Abstract

*Background*. Measures of spousal effect during parturient pain should take a tripartite approach involving the parturients, spouses, and midwives. *Aim*. To develop and validate three questionnaires measuring spousal presence in management of parturient pain in Nigeria. *Methods*. There are two phases: (1) development of questionnaires, Abuja Instrument for Midwives (AIM), Abuja Instrument for Parturient Pain (AIPP), and Abuja Instrument for Parturient Spouses (AIPS), utilizing literatures, Kuopio instrument for fathers (KIF) and expertise of health professionals, and (2) pilot study to validate the questionnaires which were administered in two hospitals in Nigeria: midwives (*n* = 10), parturients (*n* = 10), and spouses (*n* = 10).  *Results*. Internal consistency for the three questionnaires indicated Cronbach's alpha coefficient of 0.789 (AIM), 0.802 (AIPP), and 0.860 (AIPS), while test-retest reliability was *r* = 0.99 (AIM), *r* = 0.99 (AIPP), and *r* = 0.90 (AIPS). *Conclusions*. AIM, AIPP, and AIPS provide a means of investigating the effectiveness of spousal presence in management of parturient pain in Nigeria. However, further testing of each instrument is needed in a larger population to replicate the beneficial findings of AIMS, AIPP, and AIPS which can contribute rigor to future studies.

## 1. Introduction

Pain in childbirth may be one of the most excruciating experiences any woman may ever encounter [1]. It is a relative, subjective, and multifactorial experience influenced by cultures, previous pain events, beliefs, moods, and inherent ability to cope. Further, the International Association for the Study of Pain (IASP) Taxonomy defines pain as an unpleasant sensory and emotional experience associated with actual or potential tissue damage or described in terms of such damage [2].

Childbirth pain whether triggered by the medical or nonmedical causes can make women feel uncomfortable and anxious and become sleepless and agitated [3]. Such pain also stimulates the sympathetic nervous system which causes increase in the heart rate, blood pressure, sweat production, endocrine hyper function, and delays in prognosis [4]. As such, a pharmacological or nonpharmacological intervention of a sort is required to alleviate parturient pain [5].

A review of previous research from 2002 to 2014 reveals numerous studies supporting the positive impact of spousal presence during labor and delivery in both developed and developing countries [6–9]. Women have been seen to express comfort from the presence of a spouse thereby taking control during birth [10]. Furthermore, the most preferred choice of support for most women during delivery is their spouse [11, 12] and as such, majority of women have reported a positive birth experience with the presence of their spouses [7]. Spousal presence during childbirth is also instrumental in relieving the distress associated with uncertainty and anxiety faced by parturients when they feel physically and psychologically vulnerable [6, 13]. Additionally, there are enormous benefits accruing from spousal support during childbirth including emotional comfort, improved family communication, bonding, pain relief without analgesia, and positive birth experience [14].

The issue of parturient pain and its alleviation through nonpharmacological methods is very limited in Nigeria and remains an area that is underresearched. Nigeria is a low-income country with a patriarchal society, where pregnancy and childbirth are regarded as exclusive women's affairs. In Nigeria, previous studies mainly focus on men's participation in childbirth [15, 16]. Other international studies focus mainly on father's birth experiences [6, 7, 17] or questionnaires and instrument development such as the Kuopio instrument for fathers (KIF) [18] and the Fear of Birth Scale [19].

Arguably, no study has been conducted with focus on spousal presence as a nonpharmacological pain management intervention. In addition questionnaires with tripartite focus of evaluating the perception of parturient, their spouses, and the midwives regarding spousal presence in parturient pain management have not been developed and validated. This pilot study is centered on spousal presence as a nonpharmacological intervention for parturient pain management and development of questionnaires, focusing on the perceptions of the midwives, parturients, and their spouses. 

*Aim*. The aim of this pilot study is to develop and validate three questionnaires for measuring spousal presence in management of parturient pain in Nigeria: Abuja Instrument for Midwives (AIM), Abuja Instrument for Parturient Spouses (AIPS), and Abuja Instrument for Parturient Pain (AIPP).

## 2. Methods

This is a prospective study divided into two phases.

### 2.1. Phase 1: Development of Questionnaires

The item development of two of the questionnaires, Abuja Instrument for Midwives (AIM) and Abuja Instrument for Parturient Pain (AIPP), was developed from a prestudy review of literatures of spousal participation during parturiency [6, 10, 14–16, 20] and the third questionnaire, the Abuja Instrument for Parturient Spouses (AIPS), was derived from modifying the English version of the Kuopio instrument for fathers (KIF) which was developed by Sapountzi-Krepia and colleagues [18] after obtaining consent of the authors. The multidimensional questionnaires for this pilot study targeted spousal presence as an intervention for parturient pain from a tripartite perspective: the parturients, their spouses, and midwives. 27 items were generated for AIPP, 24 items for AIPS, and 25 items for AIM (Table 1).

### 2.2. Face and Content Validity

A panel of three experts, a professor of nursing science with extensive research and clinical expertise in maternity care, a doctor of nursing science with longstanding clinical expertise in childbirth, and a senior researcher of public health science specifically in clinical and research environment, were contacted to assess content validity of the questionnaires for relevance and clarity. The three questionnaires were sent through email to the experts and items were rated. Feedback from experts clarified appropriate use of terminologies such as “spouse instead of wife.” Further, changes were made to the terminology “pagan to traditional religions.”

### 2.3. Pretest of Questionnaires

A pretest was done among two parturients, two spouses, and two midwives. The research assistant duly informed participants that the questionnaires were at the stage of development and that their help was needed to improve the understanding of the questionnaire. AIPP and AIPS (interview checked questionnaires) were administered to the parturients and spouses, respectively, by an interviewer who gave verbal instructions to participants on the administering process. Both questionnaires evaluated the experience of parturient pain and spousal presence during parturiency in each respective group. Furthermore, AIM (self-administered questionnaire) with a written instruction on completion of the questionnaire on the first page was filled by the midwives evaluating their perception of spousal presence as a nonpharmacological intervention for parturient pain.

AIM, AIPS, and AIPP questionnaires were all completed in about 10 minutes by the parturients, spouses, and midwives. Following completion of questionnaires, each participant was asked series of questions by the researcher regarding potential difficulties with the questionnaires, such as ambiguity of words, misinterpretation of questions, inability to answer a question, sensitivity of questions, and any other perceived problems associated with the questionnaires and administration process. Feedback obtained from participants was shared with the researcher and improvement was made on questionnaire administering protocol (e.g., the interviewer should give participants adequate time to respond before next question). There were no modifications to all three questionnaires as participant's remarks regarding the questionnaires were positive.

### 2.4. Phase 2: Pilot Studies

#### 2.4.1. Data Collection

A pilot study to validate the questionnaires was carried out in June 2014 in two tertiary hospitals in Abuja, Nigeria: Wuse General Hospital and Kubwa General Hospital. The maternal delivery rate of each hospital used for this study is estimated at 2,500–3,000 births annually. Participants for the study were selected through convenience sampling. Consenting parturients were included in the study if they were within 18–35 years of age, if they had singleton pregnancy at full term gestation, and if they were within 24–48 hours after delivery. Parturients were excluded if they had caesarean section, if they were on pain medication, and if they were mentally incapacitated. The spouse's inclusion in the study was based on parturient eligibility and consenting couple. Also only midwives that were licensed by the Nursing and Midwifery Council of Nigeria were included in the study.

The three different questionnaires were administered to the respective groups of consenting participants: midwives (*n* = 10), AIM; parturients (*n* = 10), AIPP; and spouses (*n* = 10), AIPS. While AIM was self-administered and completed by the midwives, AIPP and AIPS were administered through interviews by independent inspectors. The various questionnaires were collected after each administering period. Furthermore, the three questionnaires were readministered to five of the consenting participants for test-retest reliability after five days for each group. Each questionnaire was evaluated separately for validity and reliability.

#### 2.4.2. Data Analysis

Statistical analyses were performed using the Statistical Package for the Social Sciences for Windows (SPSS 19). Respondent's demographics from the three questionnaires are described with the statistical median. Cronbach's alpha statistic was used to evaluate the reliability and Spearman's correlation among items for construct validity using the numerical variables in the questionnaires. The item analysis was considered satisfactory if Cronbach's alpha value was 0.7 or above [21]. The two-way random effects model was used for Intraclass Correlations (ICC) measure based on consistency with 95% confidence interval (CI). Correlation coefficient was used to measure the variation between the responses of five respondents for each questionnaire, respectively, who were tested twice (i.e., the test-retest sequence). A value of one indicates perfect correlation between the two responses, while a value of zero indicates no correlation between the two sets of answers [22]. The correlation coefficient values were averaged over all respondents and were repeated for all the respondents. Additionally, Spearman's correlation coefficients were calculated between items in the respective questionnaires to assess construct validity.

## 3. Results

### 3.1. Characteristics of Participants

#### 3.1.1. AIM

The median age of midwives was 33 years (IQR = 28–38) and all midwives were females. Four of the respondents were from Yoruba ethnicity, while others were (three respondents) from Igbo and Hausa (one respondent) and two respondents were from minority ethnic groups (Kaduna and Igala). Educational level shows that eight of the respondents completed general nursing school, one respondent attended vocational nursing school, and one respondent attended university. The mean average for work experience was M = 9.86 (SD 6.83) (Table 2).

#### 3.1.2. AIPP

The median age of parturients was 27.50 years (IQR = 23–32). Four participants were of Igbo ethnicity, four participants were from minority ethnicity, and the remaining two participants were of Hausa ethnicity. Educational levels indicated that five respondents only had secondary school education and one respondent attended vocational school, while four respondents had a B.S. degree. Marital status showed that nine respondents were married and one respondent was divorced. In terms of religion, four respondents were Christians, four respondents embraced traditional religions, and two respondents were Muslims (Table 2).

#### 3.1.3. AIPS

The median age of spouses was 32.50 years (IQR = 28–37). Six of the participants were of Igbo ethnicity, two participants were from minority ethnicity, one participant was of Hausa ethnicity, and one participant was of Yoruba ethnicity. Five of the respondents had a B.S. degree, two respondents had secondary education, one respondent completed vocational school, and one respondent had an M.S. degree, while one respondent had no formal education. Nine respondents are fully employed, while one respondent is unemployed. Marital status showed that nine respondents were married and one respondent was divorced. All respondents were Christians (Table 2).

### 3.2. Reliability Results for Questionnaires AIM, AIPP, and AIPS

#### 3.2.1. AIM

Two domains, (a) midwives pain management practices and (b) perception of spouse's presence during parturiency, with four and two variables, respectively, had Cronbach's alpha coefficient of 0.789 and 0.780 (Table 3). The average measure for ICC was in the two domains (a) 78.9% and (b) 78% with 95% CI of 0.415–0.947 and 0.116–0.947 showing an acceptable degree of reliability. The total mean for AIM variables in both domains was M = 6.81; SD = 2.11.

#### 3.2.2. AIPP

Selected six items on AIPP domain assessing parturient pain alleviation relating to spouses participation in childbirth had Cronbach's alpha coefficient of >0.802 (Table 3). The correlation coefficient for test-retest reliability *r* = 0.99. The average measure for ICC was 80% with 95% confidence interval of 0.522–0.943 showing an acceptable degree of reliability. The total mean for AIPP variables was M = 14.40; SD = 3.062.

#### 3.2.3. AIPS

All nine factors in AIPS domain evaluating spouses participation and perception of spouses as an intervention of alleviating parturient pain had Cronbach's alpha coefficient of >0.86. Internal consistencies for the following AIPS items showed births attended B3 (0.701), presence importance B7 (0.713), spouses pain B8 (0.763), and presence alleviated pain B9 (0.885), while number of births B1 (0.540) and rate spouses pain B10 (0.603) were low, respectively. Though two selected items in AIPS had rather low alpha and the overall alpha score would have increased if these items were deleted, they were retained due to minimal impact their deletion will have on the increase of the overall score. The items were retained. Further, correlation coefficient for test-retest reliability *r* = 0.90. The average measure for ICC was 0.860 with 95% CI of 0.860–0.956 showing an acceptable degree of reliability (Table 3). The total mean for all nine variables of AIPS was M = 24.30; SD = 5.85.

### 3.3. Correlation Results on Item Scores in Respective Questionnaires

Table 4 illustrates Spearman's rho on AIM items; pain assessment, pain relief necessary, and pain intervention showed a good correlation (rho = 1.0; *n* = 10; *P* < 0.001). Perception on spouse contribution to pain alleviation and encouraging spouse presence during childbirth also indicated a positive correlation (rho = 0.67; *n* = 10; *P* = 0.35).

Table 5 shows correlation results on AIPP items; spousal presence and pain relief showed a relationship of positive correlations (*r* = 1.0; *n* = 10; *P* < 0.001), while correlations between items, spouse relief and rating pain, after spousal intervention had a negative correlation (*r* = −0.80; *n* = 8; *P* < 0.17).

AIPS items Spearman's rho correlations are shown in Table 6. Perception of spousal importance during childbirth and spousal presence contributing to parturient pain alleviation showed a positive relationship (*r* = 1.0; *n* = 8; *P* < 0.001).

Spearman's correlation between items scores on the three questionnaires, respectively, indicated moderate relationships between selected variables: |.667–1.0| in AIM, |.333–1.0| in AIPP, and |1.0| in AIPS.

## 4. Discussion

This study developed and validated three questionnaires, Abuja Instrument for Midwives (AIM), Abuja Instrument for Parturient Pain (AIPP), and Abuja Instrument for Parturient Spouses (AIPS), to evaluate the use of spousal presence in management of parturient pain in Nigeria from a tripartite approach. All questionnaires administered were filled and returned. This demonstrates a perceived importance for development of these questionnaires. For face and content validity, averagely, over 95% of the answers by respondents corresponded to questions on the three questionnaires, indicating a high level of understanding and clarity of questionnaire content by respondents.

Further, response from participants assisted in providing appropriate answers for the subject under study and answers were short and precise, thus enabling the researcher to quickly understand the mindset of the respondents especially with AIPP and AIPS which were administered through interview. The level of parturient pain in childbirth was easily indicated on the Universal Pain Assessment Scale added to AIPP and AIPS questionnaires as a pain measuring tool by all respondents whose responses were dependent on the facial expression recalled from the parturition period or experience. All three questionnaires presented good content validity.

Construct validity is the degree to which a test measures what it claims or purports to be measuring [23]. Results are indicative that Spearman's rho from AIM items shows a relationship between increase in parturient pain assessment and necessity for pain relief and an increase in parturient pain management interventions such as spousal presence. It demonstrated the questionnaire measuring the midwifery parturient pain practices and further evaluating the midwife's perception on spousal presence as a parturient pain management intervention.

Further, AIPP items, spousal presence and pain relief, showed a relationship of positive correlations, while correlations between items, spouse relief and rating pain after spousal intervention, had a negative correlation. Apparently, parturient pain relief increases with the presence of spouses and the inverse relationship between the spouse relief and rating of parturient pain is indicative of parturient pain reduction after spousal presence. The impact and effects of spousal presence are measured in terms of spouse relief by rating parturient pain before and after the intervention. This is suggestive that the questionnaire is measuring spousal presence as an intervention for relieving parturient pain from the perspective of the parturient.

On the other hand, AIPS items, perception if spouse presence was important and contribution to parturient pain alleviation, showed a positive relationship. The values of the correlation can be indicated as large going by Cohen guidelines where Spearman's correlation value of 0.5 is considered large [24]. Spearman's correlations show good relationship between items on AIM, AIPP, and AIPS measuring spousal presence as a parturient pain management intervention, thus justifying good construct validity.

There was an acceptable internal consistency of items on each of the questionnaires, with Cronbach's alpha coefficient of >0.70 (AIM, *α* = 0.78, AIPP, *α* = 0.80, and AIPS, *α* = 0.86) which is the cutoff value for being acceptable [23], notwithstanding that very high Cronbach's alpha would indicate redundancy of items on the questionnaires [25]. Test-retest reliability of AIM, AIPP, and AIPS on half of the respondents who completed the questionnaires twice showed that over 96% of questions were answered correctly. The answers from each respondent's questionnaires were compared and this was done for all the three groups of questionnaires. The responses were similar in all questions. However, slight discrepancies were recorded, especially in the sections where open-ended questions with multiple responses were present. Two of the questions in AIM had more responses and one question from AIPP recorded responses in slightly different order from the previous questionnaire. Repeatability for AIM, AIPP, and AIPS between the first and second application of questionnaires showed good repeatability for selected items on parturient pain assessment.

AIM, AIPP, and AIPS are new questionnaires for gathering information from midwives, parturients, and spouses, respectively, assessing knowledge, practices, perceptions, and efficiency of spousal presence in the management of parturient pain. Utilization of these questionnaires promotes a holistic approach of collecting information. Thus it is recommended that the application of these questionnaires in future studies will enhance a better understanding of practices and challenges arising from diverse views on the subject.

## 5. Limitations

The findings of this study need to be replicated in larger samples of participants (parturients, spouses, and midwives) due to a small sample size and sample selection was based on convenience sampling method.

## 6. Conclusion

This study attempted to provide three valid and reliable questionnaires in assessing spousal presence in management of parturient pain for use in a Nigerian context. Findings in this study are indicative that the three questionnaires are perceived as being acceptable, simple, and clear to all participants, attributed to a result of low missing data. Further, the questionnaires had introductory evidence for internal consistency, test-retest reliability, and validity. As such AIM, AIPS, and AIPP provide an objective means of evaluating the use of spousal presence in management of parturient pain in Nigeria. However, further testing of each instrument is needed in a larger population to replicate the beneficial findings of AIMS, AIPP, and AIPS which can contribute rigor to future studies.

## Figures and Tables

**Table 1:** Description of the questionnaires, Abuja Instrument for Midwives (AIM), Abuja Instrument for Parturient Pain (AIPP), and Abuja Instrument for Parturient Spouses (AIPS).

Questionnaire	Number of items	Questionnaire layout	Questionnaire objective	Questionnaire format
AIM	20	Three sections: (a) Demographics (8 items) (b) Pain assessment and intervention (8 items) (c) Feelings and attitude relating to spouses presence (4 items)	To evaluate parturient pain management practices and perception of the use of spousal presence as intervention by midwives.	Open questions (6) Close-ended questions (14)

AIPP	27	Three sections: (a) Demographics (5 items) (b) Birth history and parturient pain (17 items) (c) Perception of spousal presence during parturiency (5 items)	To assess spousal presence in alleviation of parturient pain and the perception of the parturient on the use of this intervention.	Open questions (5) Close-ended questions (17) Likert scale of 5 points (3) Universal Pain Assessment Scale (2)

AIPS	24	Three sections: (a) Demographics (7 items) (b) Labor and pain management (10 items) (c) Feelings and perception related to spouse labor and pain (5 items)	Assessment of spouse's participation during parturiency and their perception of parturient pain and being present during parturiency.	Open questions (5) Close-ended questions (13) Likert scale of 5 points (3) Universal Pain Assessment Scale (1)

**Table 2:** Characteristics of participants.

Variables	Midwives (*n* = 10)	Parturient (*n* = 10)	Spouses (*n* = 10)
Age			
20–24	2	3	—
25–30	3	4	1
31–35	—	3	5
35–40	4	—	3
41–45	1	—	1
Gender			
Male	—	—	10
Female	10	10	—
Educational qualification			
Secondary school	—	5	2
Vocational school	—	1	1
Vocational nursing school	1	—	—
General nursing school	8	—	—
University (B.S. or M.S.)	1	4	6
Occupation			
Nurse/midwife	10	—	—
Accountant	—	—	1
Lawyer	—	—	1
Driver	—	—	1
Sociologist	—	—	1
Civil servant	—	—	2
Architect	—	—	1
Artist	—	—	1
Businessman	—	—	2
Midwifery professional position			
Registered nurse/midwife	2	—	—
Nursing officer II	4	—	—
Principal nursing officer	2	—	—
Matron	2	—	—
Midwifery work experience (years)			
1–5	4	—	—
6–10	1	—	—
11–15	2	—	—
16–20	3	—	—
Ethnicity			
Igbo	3	4	6
Yoruba	4	—	1
Hausa	1	2	1
Minority	2	4	2
Religion			
Christian	10	4	10
Muslim	—	2	—
Traditional religion	—	4	—
Marital status			
Married	10	9	9
Divorced	—	1	1

**Table 3:** Reliability results for Abuja Instrument for Midwives (AIM), Abuja Instrument for Parturient Pain (AIPP), and Abuja Instrument for Parturient Spouses (AIPS).

Questionnaire	Reliability coefficients	Alpha standardized item	Alpha Intraclass Correlation coefficients (95% CI)
AIM			
A	0.789	.818	78.9 (.415–.947)
B	0.780	.800	78 (.116–.945)
AIPP	0.802	.827	80 (.522–.943)
AIPS	0.860	.936	86 (.679–.959)

*Note*. Cronbach's alpha value *α* = 0.7 or above.

**Table 4:** Spearman's rho items correlations of Abuja Instrument for Midwives (AIM).

		1	2	3	4	5
(1) Relief necessary	rho	1.000				
	Sig.	—				
	*n*	10				

(2) Assess pain	rho	1.000^*∗∗*^	1.0			
	Sig.	—	—			
	*n*	10	10			

(3) Interventions for pain	rho	1.000^*∗∗*^	1.000^*∗∗*^	1.000		
	Sig.	—	—			
	*n*	9	9	9		

(4) Spouse presence to pain relief	rho	−.667^*∗∗*^	−.667^*∗∗*^	−.661	1.000	
	Sig.	.035	.035	.052	—	
	*n*	10	10	9	10	

(5) Encourage spouse presence	rho	1.000^*∗∗*^	1000^*∗∗*^	1000^*∗∗*^	.667	1.000
	Sig.	—	—	—	.035	—
	*n*	10	10	9	10	10

rho, Spearman's rho; Sig., significant at 0.05 level^*∗*^ and at 0.01 level^*∗∗*^.

**Table 5:** Spearman's rho items correlations of Abuja Instrument for Parturient Pain (AIPP).

		1	2	3	4
(1) Spouse presence importance	rho	1.000			
	Sig.	—			
	*n*	10			

(2) Spouse relief	rho	−.333	1.000		
	Sig.	.420	—		
	*n*	8	8		

(3) Spousal presence	rho	1.000^*∗∗*^	−.333	1.000	
	Sig.	—	.420		
	*n*	10	8	10	

(4) Rate pain after intervention	rho	0.47	−.800^*∗∗*^	0.47	1.000
	Sig.	.879	.017	.879	—
	*n*	10	8	10	10

rho, Spearman's rho; Sig., significant at 0.05 level^*∗*^ and at 0.01 level^*∗∗*^.

**Table 6:** Spearman's rho items correlations of Abuja Instrument for Parturient Pain (AIPS).

		1	2
(1) Presence importance	rho	1.000	
	Sig.	—	
	*n*	10	

(2) Spouse presence in alleviating pain	rho	1.000^*∗∗*^	1.000
	Sig.	—	—
	*n*	10	10

rho, Spearman's rho; Sig., significant at 0.01 level^*∗∗*^.
